# Understanding the influence of surgical parameters on craniofacial surgery outcomes: a computational study

**DOI:** 10.1098/rsos.231158

**Published:** 2024-04-03

**Authors:** K. H. He, J. L. Bruse, N. Rodriguez-Florez, D. Dunaway, O. Jeelani, S. Schievano, A. Borghi

**Affiliations:** ^1^ Ningbo University, Ningbo, People's Republic of China; ^2^ Great Ormond Street Institute of Child Health, University College London, London, UK; ^3^ Institute of Cardiovascular Science, University College London, London, UK; ^4^ Vicomtech Foundation, Basque Research and Technology Alliance (BRTA), San Sebastian, Spain; ^5^ Universidad de Navarra, TECNUN Escuela de Ingenieros, San Sebastian, Spain; ^6^ Ikerbasque, Basque Foundation for Science, Bilbao, Spain; ^7^ Craniofacial Unit, Great Ormond Street Hospital for Children, London, UK; ^8^ Department of Engineering, Durham University, Durham, UK

**Keywords:** craniosynostosis, finite-element modelling, pre-operative planning, design of experiments, craniofacial surgery

## Abstract

Sagittal craniosynostosis (SC) is a congenital condition whereby the newborn skull develops abnormally owing to the premature ossification of the sagittal suture. Spring-assisted cranioplasty (SAC) is a minimally invasive surgical technique to treat SC, where metallic distractors are used to reshape the newborn’s head. Although safe and effective, SAC outcomes remain uncertain owing to the limited understanding of skull−distractor interaction and the limited information provided by the analysis of single surgical cases. In this work, an SC population-averaged skull model was created and used to simulate spring insertion by means of the finite-element analysis using a previously developed modelling framework. Surgical parameters were varied to assess the effect of osteotomy and spring positioning, as well as distractor combinations, on the final skull dimensions. Simulation trends were compared with retrospective measurements from clinical imaging (X-ray and three-dimensional photogrammetry scans). It was found that the on-table post-implantation head shape change is more sensitive to spring stiffness than to the other surgical parameters. However, the overall end-of-treatment head shape is more sensitive to spring positioning and osteotomy size parameters. The results of this work suggest that SAC surgical planning should be performed in view of long-term results, rather than immediate on-table reshaping outcomes.

## 1. Introduction

Craniosynostosis consists of premature fusion (ossification) of one or more cranial sutures during infancy, leading to predictable alterations in cranial morphology [1]; it affects 5.9 per 10 000 live births [2]. The most common presentation is scaphocephaly (long and narrow head shape), caused by sagittal craniosynostosis (SC), which occurs when the sagittal suture fuses. SC causes aesthetic [3] and—in 24% of the population [4]—functional problems. Conventional treatment for this condition involves the surgical removal of the fused sagittal suture, placement of several cuts along the skull bones to allow for appropriate brain growth and, in some instances, customized post-surgery moulding helmet therapy for 4–6 months. The surgery usually takes 4–5 h, and complications are mainly related to bleeding and the need for a transfusion [5].

**Figure 1.**
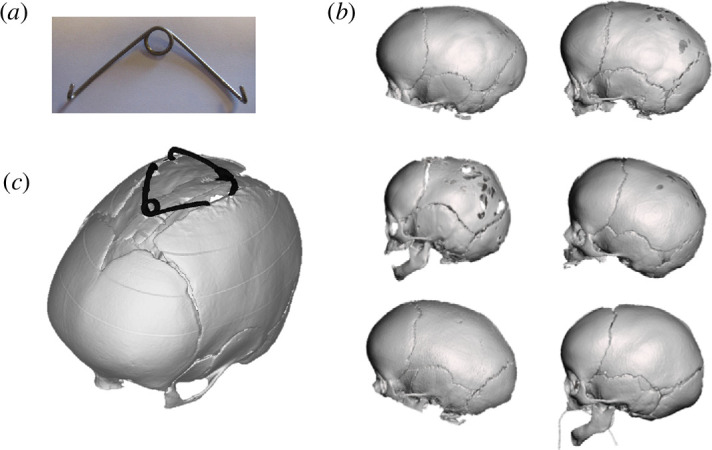
(*a*) Example of cranioplasty spring. (*b*) Three-dimensional computed tomography reconstruction of sample patients from the population used in this study. (*c*) Example of the three-dimensional reconstruction of a patient’s skull after spring-assisted cranioplasty surgery.

A novel approach for treating scaphocephaly is using metallic distractors, called springs (spring-assisted cranioplasty (SAC); figure 1*a*), which are inserted surgically by an expert craniofacial surgeon in the patient’s skull after two bony cuts are made to free the fused suture (figure 1*c*). Over the weeks following insertion, the springs gradually expand, allowing for a slow but effective remodelling of the cranial vault [6] (figure 1*c*). Springs (three different wire diameters resulting in three different stiffnesses) have been used at Great Ormond Street Hospital for Children (GOSH) since 2008 [7] and shown to be extremely safe [7], effective [6] and consistent [8]. SAC is a less-invasive procedure compared with conventional surgery, resulting in lower blood transfusion rates and shorter post-operative intensive care unit stays, and also when compared with endoscopic strip craniectomy with post-operative moulding helmet therapy [9].

SAC is gaining popularity in the craniofacial surgical field but is still at times criticized by the surgical community because of the need for a second operation to remove the springs, the lack of expansion control and the limited understanding of the spring–skull interaction. Recent works from our group showed that the spring and skull interact in a specific mechanical manner [10], with the latter responding viscoelastically to the forces exerted by the distractors. Our previous works have demonstrated that the opening of distractors follows an exponential growth pattern, with an average time constant of 1.16 days [10], which is in line with the values of bone relaxation time constants [11] estimated using material optimization against retrospective clinical spring measurements. Within an SAC population, forces completely dissipate within 10 days from implantation. Our group produced and validated a finite-element (FE) model based on a population of real patient anatomies that can predict the amount of spring expansion as well as the shape of the head post-expansion [11] within 5% error. Further validation in a larger cohort [12] and expansion to different craniofacial procedures [13,14] showed that FE modelling is a robust tool for assessing and predicting the skull response to spring distraction forces and is ready to be used for surgical planning.

Patient-specific modelling provides important information on spring dynamics but does not allow for the generalization of patient results. A recent development in surgical research has focused on the adoption of statistical shape modelling to describe the shape variability and its effect on surgical outcomes [15]. This methodology has proved extremely powerful in describing anatomical variations in a population of shapes [15–20], as well as the effect of surgical outcomes [6,21–23]. Integration of statistical shape modelling and FE modelling has been attempted in the past for analysing the sensitivity of cranial stress patterns due to physiological loads in primates [24] and rodents [25], as well as for analysing the phenomenon of natural adaptation in insects [26] and amphibians [27].

In this work, an average model of a scaphocephalic skull was created by means of statistical shape modelling and used to simulate the on-table effect of spring insertion over time via finite-element analysis (FEA). Surgical parameters were varied to assess the effect of spring positioning and combinations on skull shape and dimensions.

## 2. Methodology

### 2.1. Patient populations

The GOSH Craniofacial Unit patient database was reviewed, and the retrospective imaging data (computed tomography (CT) scans, X-ray imaging and three-dimensional scans) from three distinct patient groups (previously used in other studies by our group) were included in this study.

Thirteen SAC patients (11 male and age at SAC = 5.6 ± 0.7 months (4.6–6.7 months)), operated on between 2011 and 2016 and presenting with pre-operative CT scans, were included in this study. Each patient had a pre-operative CT scan performed between 3.2 months and 6 days earlier (age at CT scan = 4.2 ± 0.7 months (3.0–5.3 months); table 1). CT scans were acquired using standard ‘head CT’ protocols (with the patient wrapped up and in the supine position) at different institutions and transferred to GOSH prior to surgical intervention. In-plane resolution was 0.38 + 0.05 mm (range 0.30–0.47 mm), and slice thickness was 0.81 + 0.26 mm (range 0.5–1.25 mm).

**Table 1.** Patient groups.

imaging used	CT scans (template model)	three-dimensional scans (validation)	X-ray (validation)
number of patients	13	20	42
age of the population	5.6 ± 0.7 months	5.1 ± 1 month	5.3 ± 0.9 months

To compare the results found in this study with the outcome of actual surgery, retrospective clinical patient scans (X-ray imaging and three-dimensional stereophotogrammetry; table 1) were processed. Planar X-ray images for a group of patients (age at X-ray scan = 5.3 ± 0.9 months, *n* = 42; table 1), having suitable pre-operative and follow-up imaging (at day 1 and 3 weeks of follow-up) and for whom information on the spring model had been recorded, were collected.

**Table 2.** Spring models with constants.

model	longitudinal stiffness, K (N/mm)	free length, OP∞ (mm)
S10	0.17	60.7
S12	0.39	57.3
S14	0.68	55.6

Similarly, three-dimensional scans of a group of patients who had received on-table three-dimensional stereophotogrammetry scans pre-operatively and at the time of removal, for whom information on the osteotomies was available (age at the first three-dimensional scan = 5.1 ± 1.0 month, *n* = 20; table 1), were collected and processed to extract information on cranial expansion. Three-dimensional scans were acquired using a structured light three-dimensional handheld surface scanner (M4D Scanner, Rodin4D, Pessac, France) with the patient on-table, wearing a white nylon stocking. Three-dimensional shapes were processed to remove artefacts and fill in holes. Details on the three-dimensional scan acquisition and processing can be found in the study by Tenhagen *et al*. [6].

### 2.2. Average skull model

**Figure 2.**
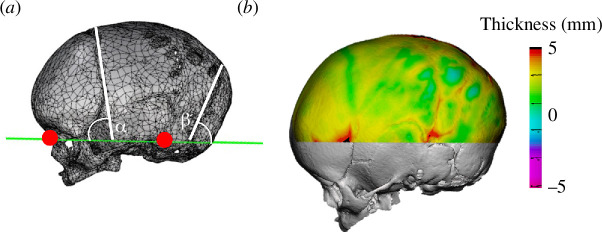
Example of (*a*) a patient‘s three-dimensional skull model reconstructed from a CT scan. The green line visualizes the position of the reference cutting plane passing through the nasion (red spot, left) and upper auditory meatus (red spot, right). (*b*) Skull thickness distribution.

The three-dimensional shape of the skull (triangular mesh) of each patient was reconstructed from CT scans using Mimics (Materialise, Leuven, Belgium). A standard bone window (with a Hounsfield unit above 226) was used for the skull segmentation in all patients, with sutures appearing as empty voids owing to the lower signal shown in the CT scans. Each three-dimensional shape was further processed in Rhino3D (McNeel, Seattle, WA, USA) and Meshmixer (Autodesk, San Francisco, CA, USA) to (i) create a reference plane through the nasion and the right and left external auditory meatuses for consistency (figure 2*a*), (ii) fill in skull defects and sutures, and obtain a smooth continuous surface, and (iii) separate the inner surface of the skull bone from the outer surface.

For each patient, the localized skull thickness was measured as the surface distance between the inner and outer surfaces, and to exclude the orbits from the thickness calculation, only the top 25% of the model height was considered (figure 2*b*). The average skull bone thickness was calculated for each patient and then further averaged throughout the patient cohort. The angles between the plane running through the coronal suture and the reference plane (*α* in figure 2*a*) and between the plane running through the lambdoid suture and the reference plane (*β* in figure 2*a*) were manually measured in each patient by visually identifying the planes running through the sutures; the values of *α* and *β* were averaged for the population.

The outer skull surfaces were aligned, and an average shape model was calculated for the population using statistical shape modelling, implemented in Deformetrica (www.deformetrica.org) [15]. The software computes the three-dimensional mean anatomical shape of a population (template shape) by minimizing the distance (in terms of a deformation vector) with each subject shape. Starting from this average shape model, a solid skull model was recreated in Solidworks (Dassault Systems, Paris, France) by offsetting internally the average model surface by the same value as the average population skull bone thickness. The function ‘offset surface’ implemented in Rhino3D, with the option ‘solid activated’, was used to create the thickness. The surface was then exported into Solidworks, and the model was split into five parts: frontal bone, coronal suture, parietal bone, lambdoid suture and occipital bone. The average values of *α* and *β* were used to simulate the location of the two sutures, assumed to be 2 mm thick [28].

### 2.3. Finite-element modelling

**Figure 3.**
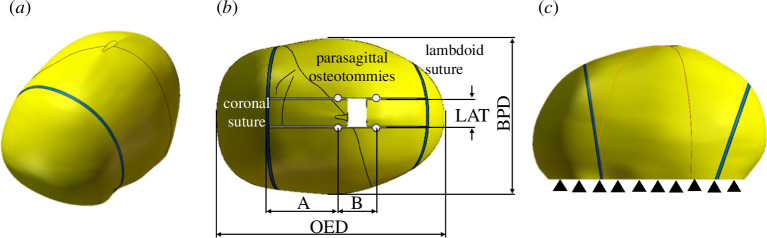
(*a*) Three-quarter view of the skull model (yellow) with sutures (blue). (*b*) Top view of the model showing surgical and craniofacial parameters: A = distance of the anterior spring from coronal suture, B = distance of the posterior spring from the anterior spring, LAT = distance between the parasagittal osteotomies, OFD = occipitofrontal diameter = skull length,; BPD = biparietal diameter = skull width. (*c*) Side view of the model, showing the constraints on the bottom border.

The framework for FE modelling of spring expansion has already been published by our group [11,12]. Briefly, parasagittal cuts were performed on the parietal section of the bone in Solidworks. Spring expansion was modelled using linear spring conditions available in ANSYS (v. 2022 R2), with each spring condition (REF ANSYS MANUAL) defined by two parameters: stiffness and free length as shown in table 2. The spring opening versus load curves were reported by Borghi *et al.* [10]. Surgical notches with a 2.5 mm radius were positioned in correspondence with the location of the springs, and a linear condition was applied between each notch pair (figure 3), with stiffness and free length chosen according to the spring model combination applied [10,11] and reported in table 2. As in our previous work, the material was modelled as viscoelastic using a Prony shear relaxation relationship

**Table 3.** Skull mechanical properties

index	relaxation relative modulus	relaxation time constant
*i* = 1	0.73213	6720.4
*i* = 2	0.25708	40322


$$\frac{G\left(t\right)}{G_{0}}=\alpha_{\infty }+\sum_{i}\alpha_{i}\cdot e^{-}\frac{t}{\tau_{i}},$$

where *α*_*∞*_ and *α*_*i*_ are the relative moduli, *t* and *τ*_*i*_ are the time constants, *G*(*t*) is the time-dependent shear modulus and *G* is the initial shear modulus. Material (elastic and viscoelastic) properties were retrieved from the previous work by our group [12] and reported in table 3.

The inferior border of the model, which lays on the plane passing through the nasion and tragions, was fully constrained in all directions to simulate the presence of the skull base and to prevent rigid translations/rotations (figure 3*b*). A mesh sensitivity analysis was performed—using the same boundary conditions and spring conditions—where the mesh density was increased until the estimated spring expansion value did not change by more than 5%.

Cranial reshaping due to spring expansion was modelled over 5 days. At selected time points (*t* = 1 s, 1 day and 5 days), the deformed model biparietal diameter (BPD) and occipitofrontal diameter (OFD) were extracted, and the cranial index (standard craniometric index (CI) used to assess craniofacial proportions) was calculated as


(2.1) $$CI=\frac{BPD}{OFD}.$$

At such time points, the increase in CI following the spring expansion was calculated as ΔCI_*t*_ = CI(*t*) – CI(*0*), where CI(*0*) was the CI of the undeformed model, the same for each surgical configuration analysed. Thus, ΔCI_post-op_ was calculated at *t* = 1 s, equivalent to immediate post-operative; ΔCI_day 1_ at day 1 follow-up; and ΔCI_follow-up_ at *t* = 5 days, equivalent to the long-term follow-up.

### 2.4. Parametric study of spring expansion

Patient details of the surgical procedure—in terms of the distance between the parasagittal osteotomies (LAT), the distance between the coronal suture and the anterior spring (*A*), and the distance between the anterior and posterior springs (*B*; figure 3) and implanted springs (stiffness of anterior *K*_ANT_ and posterior *K*_POST_ springs)—were available for eight of the patients in this study.

Each size was normalized according to the patient-specific skull dimensions, such as BPD and OFD:


(2.2) $$LAT\%=\frac{LAT}{BPD}\times 100,$$

(2.3) $$A\%=\frac{A}{OFD}\times 100,$$

(2.4) $$B\%=\frac{B}{OFD}\times 100.$$

The normalized parameter ranges were averaged throughout the population and recorded in terms of population mean ± s.d.

The effect of the variation in surgical parameters (LAT%, *A*%, *B*%, *K*_ANT_ and *K*_POST_ *—input parameters*) on ∆CI_*t*_ at the selected time points (*output parameters*) was examined using design of experiments (DoEs) implemented in ANSYS. DoE is an engineering method used to minimize the number of experiments necessary to characterize the parametric sensitivity of a mechanical system. Each parameter combination constitutes a ‘design point’, and the number of design points is calculated by the software according to the DoE type and the number of input parameters (in this case, the input surgical parameters). The optimal space-filling DoE method was adopted as this method maximizes the distance between design points to achieve a more uniform distribution across the design space; 79 configurations (= design points) were created by the software, each related to a different surgical scenario where LAT%, *A*% and *B*% were varied in the range (population mean – s.d.; population mean + s.d.) and the spring stiffnesses (*K*_ANT_ and *K*_POST_) and free length were varied within the (minimum; maximum) range as reported in the study by Borghi *et al.* [11].

Parameter sensitivity was defined as the rate of output change versus change in input parameter when all other input parameters are maintained constant at the mid-range value. The sensitivity of ∆CI (at day 1, post-operative and follow-up) to each surgical parameter was extracted from the findings and compared across the population. A positive/negative sensitivity value means that the ∆CI at the selected time point increases/decreases with the increase in the selected surgical parameter.

### 2.5. Comparison with clinical measurements

The X-ray imaging was processed: BPD and OFD were measured at each time point and CI was calculated using equation (2.1). The combined stiffness was calculated as *K*_TOT_ = *K*_ANT_ + *K*_POST_.

Three-dimensional scans were processed to extract pre-operative and end-of-treatment CI using a protocol previously published by our group [6]. The position of the anterior spring was assumed to be equal to *A*%; the position of the posterior spring was calculated as (*A* + *B*)% = *A*% + *B*%.

## 3. Results

### 3.1. Study population

The average measured skull thickness was 2.02 ± 0.24 mm. The coronal and lambdoid suture planes laid at an angle *α* = 81.5 ± 2.5° and *β* = 70.9 ± 2.5°, respectively. The measurements of OFD and BPD throughout the population were equal to 108.3 ± 4.2 mm and 152.6 ± 4.3 mm, respectively. In comparison, the average skull model calculated in Deformetrica had OFD = 105.4 mm (−2.7% difference compared with the population average) and BPD = 154.9 mm (1.5% difference compared with the population average). The average values for the surgical parameters were LAT% = 16.9 ± 2.1%, *A*% = 31.4 ± 6.5% and *B*% = 17.2 ± 6.9%.

To assess the result trends against clinical data, the ΔCI was measured from planar X-rays (available pre-operatively, at day 1 and at 3 weeks, as per clinical protocol) and three-dimensional scans (acquired pre-insertion and at removal). X-ray scans yielded the following values: ΔCI_day 1_ = 3.4 ± 2.5% and ΔCI_follow-up_ = 4.4 ± 2.5%. Three-dimensional scans yielded the following value: ΔCI_follow-up_ was 3.7 ± 1.7%.

### 3.2. Parametric study

FE spring expansion was simulated in the considered 79 design points, with each having a different set of surgical parameters.

**Figure 4.**
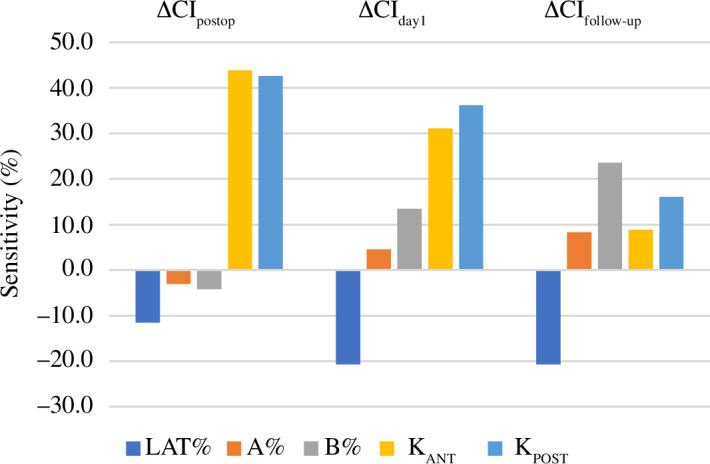
Bar chart showing the sensitivity of the increase in CI (ΔCI) at simulation times equivalent to post-operative, day 1 and follow-up.

The sensitivity of the CI change (∆CI) to the surgical parameters (LAT%, *A*%, *B*%, *K*_ANT_ and *K*_POST_) at the three time points (post-operative, day 1 and follow-up) was calculated. Immediately post-operative, the spring stiffnesses (*K*_ANT_ and *K*_POST_) have an overall more pronounced effect (43.9% and 42.6%, respectively) than the osteotomy and spring position parameters (LAT%, −11.6%; *A*%, −3.1% and *B*%, −4.2%). The ∆CI sensitivity changes at day 1 post-operative, with the spring stiffnesses having a lower sensitivity (31.1% for *K*_ANT_ and 36.2% for *K*_POST_) while the spring positioning (*A*%, 4.5% and *B*%, 13.5%) and osteotomy width (LAT%, −20.7%) have a more evident effect. At follow-up, the sensitivity to spring stiffness further decreases (8.9% for *K*_ANT_ and 16.1% for *K*_POST_) and ΔCI_follow-up_ appears to be particularly sensitive to LAT% and *B*% (−20.7% and 23.6%, respectively). Figure 4 shows the sensitivity bar chart for ΔCI.

**Figure 5.**
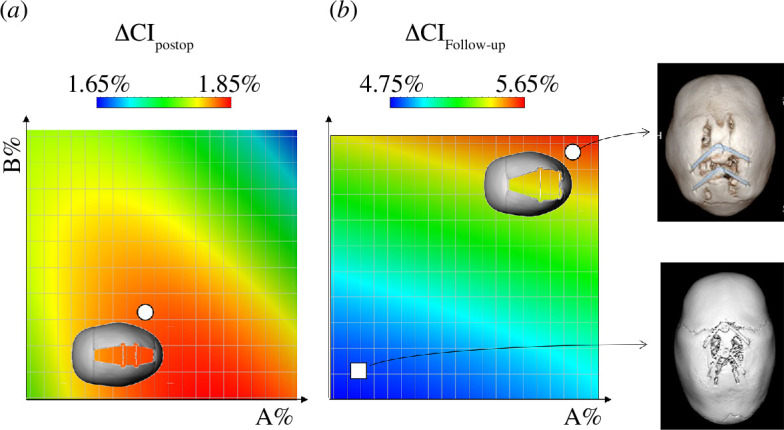
Visualization of predicted ΔCI at (*a*) post-operative and (*b*) follow-up according to the variation in surgical parameters *A*% and *B*%. The white circles show the combination of surgical parameters yielding the maximum CI increase with the relative predicted shape. On the right, examples are shown of patient outcomes where the surgical parameter selection was close to the optimal combination (circle) and suboptimal combination (square).

Figure 5 visualizes the simulated change in ΔCI according to the value of the parameters related to the spring position (*A*% and *B*%) at simulation time equivalent to post-operative (left) and follow-up (right). *A*% relates to the position of the anterior spring compared with the coronal suture; therefore, a low value of *A*% relates to a spring positioned more anteriorly, and a large value relates to a spring positioned more posteriorly. *B*% relates to the distance between the anterior and posterior springs: a low value relates to springs positioned closely, while a large value relates to springs positioned apart. Figure 5 highlights how spring position combination (i.e. combination of *A*% and *B*%) yielding a high CI change (area in red on the graphs) show differences between post-operative and follow-up. The spring position combination (in terms of *A*% and *B*%) yielding the maximum extent of head reshaping (i.e. maximum ΔCI) at post-operative (ΔCI_post-op_) and follow-up (ΔCI_follow**-**up_) was calculated and shown in figure 5, which visualizes the effect of using different combinations of spring position. The relative resulting skull shape in these two configurations was visualized and reported (figure 5).

**Figure 6.**
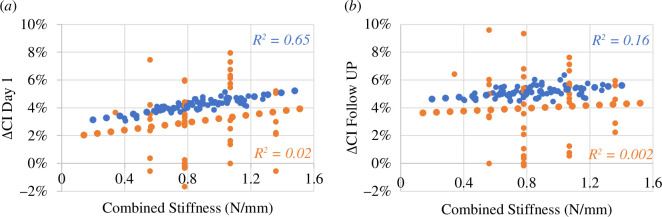
Visualization of the simulation results of the design points (in blue) versus X-ray measurements from an SAC population (orange): correlation between ΔCI and combined stiffness at day 1 (*a*) and follow-up (*b*).

The output of each design point in terms of ΔCI was compared with the direct measurements from patient X-rays to validate the effect of spring stiffness extracted from the model. Similar trends were found when analysing the relationship between combined spring stiffness and ΔCI_day1_ (figure 6*a*) as well as ΔCI_follow**-**up_ (figure 6*b*).

The output of each design point in terms of ΔCI was compared with the direct measurements from patient three-dimensional photogrammetry scans performed at the time of spring removal (+4 months after the initial surgery), to compare the effect of spring position as extracted from the model.

**Figure 7.**
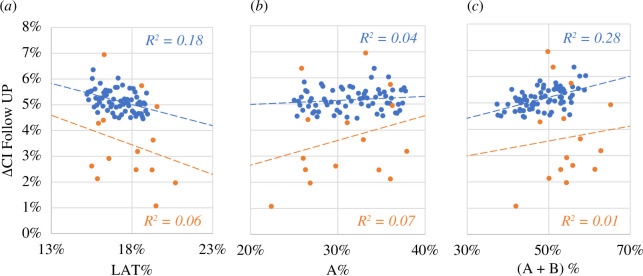
Simulation results (in blue) versus three-dimensional scan measurements from an SAC population (orange): correlation between ΔCI and follow-up and LAT% (*a*), anterior spring location (*b*) and posterior spring location (*c*).

For LAT%, a similar negative trend was found in both FEA results at follow-up and three-dimensional scan measurements at removal (figure 7*a*). The position of the anterior spring (*A*%) shows a negligible effect in the FEA extracted post-operatively, while it shows a positive trend in the measurements extracted from the three-dimensional scan (figure 7*b*); on the other hand, the position of the posterior spring (*A*% + *B*%) shows a positive trend for both groups (figure 7*c*).

The low values of *R* ^2^ for the clinical measurements in figures 6 and 7 indicate that there is a large amount of variability in the data. This could be due to individual anatomical differences among patients. It is important to note that the aim of this study is to assess the effect of surgical parameters on post-operative expansion, rather than to accurately predict clinical outcomes. The simulated data within the design space showed a pronounced correlation because it was based on an idealized model that removed the effect of anatomical variability, allowing for better visualization of trends.

## 4. Discussion

SAC is a novel procedure aimed at reshaping the skull of patients affected by SC in a minimally invasive way: biparietal widening promoted by the spring insertion causes the skull to widen over time, thus achieving a physiological value of CI and therefore normalizing the head shape [7,29–31]. The results are highly dependent on the choice of surgical parameters (craniotomy size, spring location and model), and there remains a poor understanding of calvarium–distractor interaction [31], leading to suboptimal outcomes in some cases [7].

In the current work, a combination of FE method, DoEs and statistical shape modelling was used to produce a population-averaged model of the scaphocephalic head to perform the parametric analysis of virtual spring insertion and assess the effect of surgical choices. This is an alternative approach to idealized models, widely used in orthopaedic [32] and cardiovascular [33] modelling, which generally rely on population-averaged dimensions and features with extremely simplified shapes. To create the average model, retrospective recruitment of patients who underwent CT scans between 3 and 6 months of age, which is the preferential age range for SAC according to GOSH protocol, was performed, and CT scans were processed consistently to create a template (average model) using a methodology previously developed by our group [16]. In the present study, the bone material was defined using a viscoelastic model. The material properties, including the elastic and viscoelastic parameters, were retrieved from the previous work by our group [12], which involved material optimization over a group of 18 patients. The inclusion of both instantaneous material properties (such as Young’s modulus) and population-averaged viscoelastic properties (retrieved from [12]) allowed for the simulation of both on-table expansion and long-term adaptation.

In the simulation, the spring stiffness and free length were varied, and its effects on cranial displacement over time were analysed. The gradual expansion of the springs was taken into account. Simulation results were consistent with our previous studies that found that the extent of the final spring expansion is largely independent of the adopted spring model since all distractors eventually reach over 90% of their nominal size within 3 weeks.

The results of this work suggest that while the spring distractor model (i.e. stiffness) has a pronounced effect on the skull reshaping at the time of surgery (post-operative), it becomes less important over time, and by the time expansion has terminated (follow-up), the final head shape only depends on the choice of osteotomy size (LAT) and spring position (A and B). The spring position combination that would yield the highest extent of reshaping post-operatively (spring closeby and centrally positioned) would produce suboptimal results in the long term (figure 5). On the other hand, if springs are positioned distally (one central and one close to the lambdoid suture), optimal reshaping is achieved by the time of full spring expansion. This is likely owing to the inherent asymmetry of the paediatric skull and its anatomical structure (including the coronal and lambdoid sutures), whereby a more centrally located distraction causes—upon full expansion—a more pronounced increase in height as opposed to width, which does not contribute to CI change. On the other hand, posteriorly positioned springs allow for a more pronounced increase in the BPD dimension, which affects the end-of-expansion CI. The results of this paper closely match with those reported by our group in another study [21,34], where statistical modelling was used to assess the long-term effect of spring cranioplasty on a group of patients. Our findings in that work showed that LAT, A and B have a significant effect on the final head shape. Results in such a cohort suggested that—analysed separately—a small LAT, a large value of A (centrally positioned anterior spring) and a large value of B (distally positioned posterior spring) have desirable effects on the post-explant shape. These authors also found that springs close to each other are associated with localized prominence on the top of the head at the end of the treatment, while springs further apart allow for better skull widening.

The negative sensitivity for *A*% and *B*% initially (post-operative) indicates that as the values of *A*% and *B*% increase, the cranial reshaping (ΔCI) decreases. This means that when the anterior spring is positioned more anteriorly (lower *A*%) and the distance between the anterior and posterior springs is smaller (lower *B*%), the extent of cranial reshaping is higher immediately after the surgery. However, at later time points (follow-up), the sensitivity for *A*% and *B*% becomes positive. This means that as the values of *A*% and *B*% increase, the cranial reshaping (ΔCI) also increases. In other words, when the anterior spring is positioned more centrally (higher *A*%) and the distance between the anterior and posterior springs is larger (higher *B*%), the extent of cranial reshaping is higher during the long-term follow-up period.

This change in sensitivity can be attributed to the inherent asymmetry of the paediatric skull and its anatomical structure. The initial negative sensitivity suggests that a more centrally located distraction (lower *A*% and *B*%) causes greater cranial reshaping immediately after the surgery. However, as the skull expands over time, a more posteriorly positioned anterior spring and a larger distance between the anterior and posterior springs (higher *A*% and *B*%) allow for optimal reshaping of the skull in the long term.

The conclusions reported here expand the remit of the analysis, thanks to the capability of FEA to simulate spring expansion throughout the overall treatment. The present sensitivity analysis proves that a good on-table outcome may not lead to optimal long-term results and that the final head shape is highly dependent on the choice of spring locations.

Previous works by our group showed that spring force decreases exponentially between insertion and removal [7,10,11] and that the extent of final spring expansion is largely independent of the adopted spring model since all distractors eventually reach over 90% of their nominal size within three weeks [11]. Therefore, it is understandable that surgical configurations sharing spring position but differing in spring stiffness would likely achieve the same end-of-treatment results since localized spring expansion would reach the same level by the end of the treatment. On the other hand, the choice of spring location has a mild effect on the post-insertion expansion but a far more evident effect (up to 1.9% increase in CI) on the end-of-treatment cranial shape. Figure 5 reports three-dimensional cranial reconstructions of two patients who underwent spring cranioplasty and received long-term post-operative CT scans—the final shape allows qualitative appreciation of the importance of spring positioning selection.

Other studies have attempted an analysis of the effect of surgical parameters on the outcome of SAC in other surgical centres worldwide. The results showed a lack of consistency, claiming that either age [35] or spring characteristics (spring force, spring length and duration of spring placement) [36] were predictors of the CI change. The present work shows that the spring stiffness (hence, insertion spring force) affects the final outcome; however, spring location, which was not reported in other studies, has a more evident effect and therefore should be carefully taken into account during surgical planning. The definition of spring length adopted in other centres [36] differs from that used at ours; hence, the results are not comparable. Furthermore, other centres use up to three springs to perform SAC, and this may yield a difference in the sensitivity of the distraction force. Yet, the computational framework presented here could also be translated into other surgical techniques.

This work has several limitations dictated by the specific population analysed. Patients recruited for the current study received pre-operative CT scans between the ages of 3 and 6 months. A small minority of patients who present at GOSH with the diagnosis of SC have pre-operative CT scans, most of them between 1 and 3 months of age. Since major changes occur in cranial anatomy within the first few months of life, it was deemed best to restrict the population to patients whose age was compatible with the procedure. Furthermore, to expand the remit of the comparison with clinical data trends, a combination of X-ray and three-dimensional scan measurements was used to assess the effect of spring stiffness and surgical parameters. This was necessarily owing to two factors: a change in pre-operative distractor model selection over time (over the last few years, surgeons have leaned towards the matched, mid-range stiffness, decreasing the number of spring combinations used)—which required the use of historical data (X-ray scans)—and the fairly recent inclusion of surgical parameters in the post-operative notes, which coincided with the use of on-table three-dimensional scans for outcome assessment. No information on spring location was available in the clinical records for the historical X-ray dataset, making direct comparison of patient outcomes more difficult. However, it is reasonable to assume that the surgical technique has remained similar over the years. Furthermore, both X-ray and three-dimensional scan imaging modalities have been used in the past to calculate CI and, therefore, were used in this work to assess the parametric study outcome.

Furthermore, the method used to produce average sutures using average population measurements does not consider the complex morphology and wavy pattern displayed *in vivo*, which has been proved to influence cranial strains during mastication [37]. The effect of such a complex feature on the outcome of spring cranioplasty has not been addressed yet in the literature; however, future developments of this model should take it into account.

Another limitation of the model lies in the lack of modelling of cranial growth post-insertion, which has been included in other models of craniosynostosis correction [38–41]. This feature will be included in the future development of the model; however, several works in the literature have shown that SAC patients undergo negligible CI change after the first 3–4 weeks post-surgery [7,36]; therefore, it is safe to assume that head reshaping occurs at a much faster pace than head growth.

Compared with our previous works [11,12] where up to 20 days’ spring expansion was modelled, a shorter time frame (5 days) was simulated to save computational time; however, results assessed on selected design points showed that cranial displacement had plateaued within this time frame. Furthermore, the time frame simulated is three times longer than the kinematic constant of spring opening reported in a previous study by our group (1.16 days) [10], and, therefore, it was assumed that the simulated expansion at the time point was comparable with that experienced *in vivo* after 10 days (i.e. at 3 weeks). To test this hypothesis, expansion was simulated over 20 days on 10 time points; between days 5 and 20, the CI value only increased by 0.07 ± 0.05%.

The results of this work highlight a correlation between postprocedural reshaping of the paediatric skull following SAC and surgical parameters, such as osteotomy dimension, spring location and spring model. Spring model has little influence on the final head reshaping, which is affected mostly by spring positioning. These results suggest that careful pre-operative planning should be performed by surgeons to identify the optimal location for spring insertion, while spring model selection is of secondary importance. Future works will assess population-based variations of pre-operative anatomy on the effect of SAC to allow craniofacial surgeons to further improve the outcome and safety of minimally invasive craniofacial reshaping surgeries.

## Data Availability

Data supporting this study are openly available from Dryad [42].
